# Diabetic Retinopathy–An Underdiagnosed and Undertreated Inflammatory, Neuro-Vascular Complication of Diabetes

**DOI:** 10.3389/fendo.2019.00843

**Published:** 2019-12-13

**Authors:** Stephen H. Sinclair, Stanley S. Schwartz

**Affiliations:** ^1^Sinclair Retina Associates, Media, PA, United States; ^2^Main Line Health System, Media, PA, United States; ^3^Private Practice, Main Line Health System, Wynnewood, PA, United States

**Keywords:** diabetic retinopathy, epigenetics, insulin resistance, inflammation, neurovascular, neurodegeneration, complications, microvascular

## Abstract

Diabetes mellitus is a world-wide epidemic and diabetic retinopathy, a devastating, vision-threatening condition, is one of the most common diabetes-specific complications. Diabetic retinopathy is now recognized to be an inflammatory, neuro-vascular complication with neuronal injury/dysfunction preceding clinical microvascular damage. Importantly, the same pathophysiologic mechanisms that damage the pancreatic β-cell (e.g., inflammation, epigenetic changes, insulin resistance, fuel excess, and abnormal metabolic environment), also lead to cell and tissue damage causing organ dysfunction, elevating the risk of all complications, including diabetic retinopathy. Viewing diabetic retinopathy within the context whereby diabetes and all its complications arise from common pathophysiologic factors allows for the consideration of a wider array of potential ocular as well as systemic treatments for this common and devastating complication. Moreover, it also raises the importance of the need for methods that will provide more timely detection and prediction of the course in order to address early damage to the neurovascular unit prior to the clinical observation of microangiopathy. Currently, treatment success is limited as it is often initiated far too late and after significant neurodegeneration has occurred. This forward-thinking approach of earlier detection and treatment with a wider array of possible therapies broadens the physician's armamentarium and increases the opportunity for prevention and early treatment of diabetic retinopathy with preservation of good vision, as well the prevention of similar destructive processes occurring among other organs.

## Introduction

Diabetes mellitus is a world-wide, growing epidemic with an estimated 415 million adults globally, including 30 million Americans (~9% of the US population), living with diabetes (1–4). Patients with diabetes are at increased risk of death and a myriad of serious diabetes-specific complications (neuropathy, nephropathy, retinopathy) and other associated complications or conditions with overlapping pathophysiologies (cardiovascular disease, dementia, psoriasis, non-alcoholic steatohepatitis, metabolic syndrome, and cancer (5, 6). Diabetic retinopathy is one of the most common diabetes-specific complications with an estimated global prevalence of 382 million (5, 7). In the US, almost 1/3rd of patients with diabetes over 40 years old have diabetic retinopathy with ~one in six of those with threatened vision (8).

Diabetic retinopathy is classically described by progressive alterations in the microvasculature that lead to retinal ischemia, neovascularization, altered retinal permeability, and macular edema (5). Of note, diabetic retinopathy is the leading cause of blindness in the adult working population (7, 9). Although retinopathy is common, poor patient compliance with yearly ocular screening (only 35–55% compliance) and techniques which rely on physician examination of the retina or retinal photography focused on vascular changes, detection is often delayed until after severe damage has occurred and treatments are unable to substantively restore vision (only 25–28% demonstrating improvement of ≥3 ETDRS lines) (10–15). Indeed, visual examination by optometrists or ophthalmologists detect only very poorly vascular changes (most often when they occur with intra-retinal hemorrhages) and physician examinations cannot easily or precisely define progressive changes over time. In addition, retinal photography (some with AI-assisted identification of hemorrhagic and vascular lesions) overwhelmingly only detect and define the more severe forms of retinopathy following vision loss and are poor at detecting ischemic defects of the inner retina, the exudative components of vascular leakage within the mid-retinal, and the abnormalities of the retinal pigment epithelium (5, 13, 14, 16–18).

Recent studies, however, have demonstrated that retinal neurodegeneration is a critical feature associated with the progression of the disease and that early retinal neuronal injury actually precedes microangiopathy (18–23). Indeed, all retinal layers (ganglion, bipolar, amacrine, and photoreceptor cell), demonstrate altered functions (as assessed by electroretinography and central vision analysis under reduced contrast and luminance conditions) prior to observable micropathy lesions (as assessed by fundus photography) (24–29). Therefore, defining diabetic retinopathy simply as a “microvascular complication of diabetes” is a misnomer and restricts our understanding of the condition as well as potential therapeutic approaches to address it.

The alterations in neuronal function are not likely the results of vascular injury but due to the injury of the integrated neurovascular unit (retinal neurons, and glia, along with pericytes and endothelia of the adjacent microvasculature) by direct neuroinflammatory insult that results in gradual, progressive neurodegeneration (30). Therefore, it appears more appropriate to consider diabetic retinopathy as a neurovascular degeneration rather than a pure microvascular disease (18–22). Further, the recognition that abnormalities in the neurovascular complex are likely to exist before microaneurysms or other angiopathic lesions occur suggests that specific approaches and therapies addressing this pathophysiology should be investigated and employed in the hope for major improvements in outcomes. Therefore, within the eye as well as other organs, clinically we should be referring to and studying damage to cells and tissues, rather than solely microvascular damage. For example, peripheral neuropathy is actually a function of direct damage to neurons with only a small amount of neuropathy suggested due to microvascular disease (31). Similarly, in diabetic nephropathy, typified by glomerular hyper-perfusion and renal hyperfiltration—classically signs of damage to the renal microvascular apparatus—have now been recognized to be associated with cellular damage from inflammation and apoptosis (32–34). In this same way, diabetic retinopathy is now recognized to result not only in damage to micro-vessels in the retina but to cell and tissues in the retinal neuro-vascular unit (including the glial and neuronal cells as well as the microvasculature) leading to retinal dysfunction (18–22, 29).

Although HbA1c is often cited as the strongest risk factor associated with the development and progression of diabetic retinopathy and is the primary target of most physicians, it may only account for a small (~10%) of risk while other factors are also involved (5, 35–38). Risk factors that actually cause hyperglycemia appear to be the same factors that raise the risk for retinopathy, impacting the neuronal tissue as well as causing microvascular injury. Indeed, retinopathy may be best understood in the context of a unified pathophysiologic construct of diabetes and its complications (6). This construct submits that the same pathophysiologic processes that cause injury to the pancreatic β-cell are responsible for the diabetes-specific complications of diabetes in other tissues as well as other conditions with overlapping pathophysiologies (6).

## Hypothesis

Diabetic retinopathy should be considered within the context of the β-cell centric model of diabetes whereby diabetes and its complications arise from common pathophysiologic factors that damage the β-cell—inflammation/immune regulation, the interplay of genes with environmental processes (epigenetics), and insulin resistance and abnormal metabolic environment (section Diabetes and Its Complications Arise from Common Pathophysiologies) (6, 39, 40).

### Diabetes and Its Complications Arise From Common Pathophysiologies

**Figure 1:**
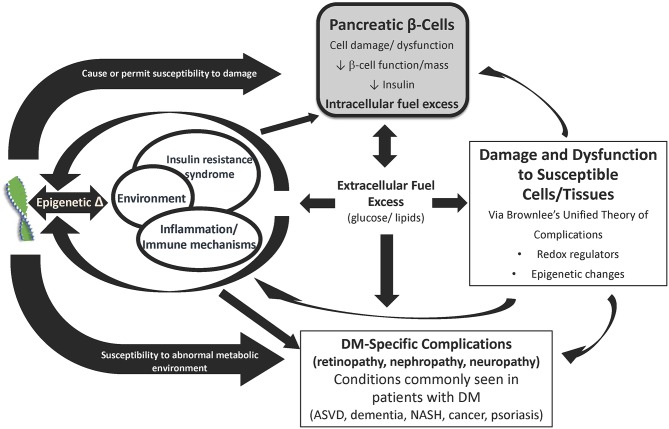
Diabetes and its complications arise from common pathophysiologies. The primary underlying mediator of diabetes complications is the damage due to hyperglycemia and other excess fuels caused by reduced insulin or reduced insulin effect. The development and progression of complications depends on tlhe interplay between genes, epigenetic changes due to the environment, insulin resistance, immune dysregulation and inflammation, fuel excess, and comorbidities (e.g., hyper tension and hyperlipidemia). ASVD, Atherosclerotic vascular disease; DM, Diabetes mellitus; IR, Insulin resistance; NASH, Non-alcoholic steatohepatitis. Source: Schwartz et al. (6). Permission for use of this figure has been obtained.

Although hyperglycemia is a core phenotype of all diabetes types, there is a single fundamental defect of the disease: pancreatic β-cell dysfunction (6). Importantly, the same pathophysiologic mechanisms that damage the β-cell also lead to cell and tissue damage causing organ dysfunction and elevate risk of developing *all* diabetes complications system wide (Figure 1). These factors (inflammation, epigenetic changes, and insulin resistance, fuel excess and abnormal metabolic environment) are responsible, to a greater or lesser degree in different individuals, for the traditional, mostly specific complications of diabetes (retinopathy, nephropathy, neuropathy, myocardiopathy), as well as other conditions frequently seen in patients with diabetes (atherosclerotic vascular disease, dementia, non-alcoholic steatohepatitis, cancer, psoriasis). β-cell dysfunction leads to an abnormal metabolic environment and the resultant fuel excess (gluco-lipotoxicity) negatively affects susceptible cells and tissues associated with diabetes-specific complications, other common conditions, as well as worsening of β-cell dysfunction.

This damage is accomplished by modulation of redox regulators and epigenetic changes in these susceptible cells and tissues that is encompassed (in part) by Brownlee's Unified Theory of “Diabetic” Complications (32, 39, 41). In essence, hyperglycemia, particularly in conditions of oscillating levels, leads to mitochondrial overproduction of superoxide that results in increased flux through four pathways—polyol, hexosamine, protein kinase C (PKC), and advanced glycation end-product (AGE) (9, 39, 42, 43). This leads to oxidative stress and reactive oxidative species (ROS) which in turn lead to inflammation and induction of transcription factors that result in altered gene expression and epigenetic changes. Ultimately, this causes cell dysfunction, hypertrophy, proliferation, remodeling, and apoptosis in susceptible cell and tissue types (e.g., β-cells, retinal cells, endothelium, neurons, vascular smooth muscle, cardiomyocytes, renal cells, etc.) (6, 39, 40, 44). Importantly, these same abnormal biochemical pathways of Brownlee's Hypothesis exacerbate the basic pathophysiologies of diabetes, its traditional, mostly specific, complications (e.g., retinopathy, nephropathy, neuropathy), and other conditions associated with diabetes (e.g., atherosclerotic vascular disease, dementia, non-alcoholic steatohepatitis, psoriasis, etc.) (6, 39, 40, 44).

**Figure 2:**
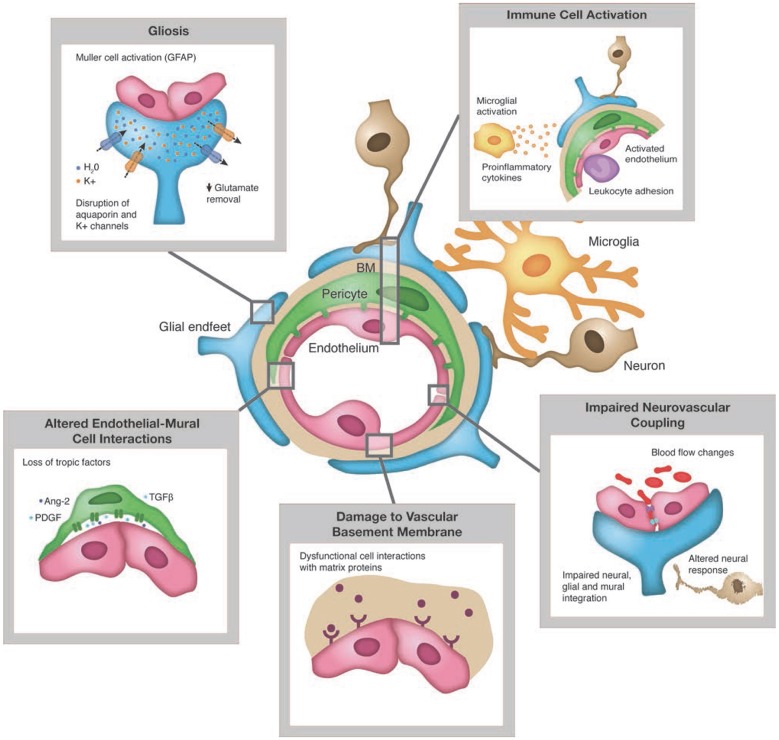
Pathologic changes to retinal neuro-vascular unit in diabetes. Source: Duh et al. (5). Permission for use of this figure has been obtained.

In the case of diabetic retinopathy, cell and neuronal tissue damage in the retinal neurovascular unit leads to glial, neural, and microvascular dysfunction—interdependent and essential factors leading to the development of diabetic retinopathy (5, 18, 20) (Figure 2). This way of thinking of diabetic retinopathy as influenced by the same pathophysiologic mechanisms driving β-cell damage as well as other complications opens up the potential of preventing, treating, or delaying retinopathy with agents used for glycemic control that also have pleotropic effects on extra-pancreatic tissues via targeting mechanisms contributing to complications (e.g., SGLT-2s on renal disease, GLP-1 agonists on cardiovascular disease) as well as agents aimed specifically at pathophysiologic mechanisms driving diabetes complications (e.g., inflammation, insulin resistance, etc.) (44, 45).

### Retinopathy Evidence Organized by Pathophysiologic Mechanism

#### Inflammation/Immune Regulation

There is growing consensus regarding the key role of inflammation in pathogenesis of diabetic retinopathy (9, 45–47). The retina is the most metabolically active tissue in the body making it very susceptible to oxidative stress both from light-induced electron injury and oxygen free radical production leading to increased inflammation (48). Indeed, diabetes and its associated hyperglycemia, insulin resistance, dyslipidemia, etc. all lead to altered biochemical pathways (polyol, AGEs, PKC, hexosamine, and renin-angiotensin system) that stimulate glial cell dysfunction (9, 39, 46, 49). This dysfunction leads to increased inflammatory cytokines and chemokine production, aberrant growth factor signaling and ROS resulting in neuro-glial degeneration and vascular dysfunction and its associated alteration of the blood-retinal barrier, hypoxia, vascular permeability resulting in edema and angiogenesis (9). This ultimately leads to the development and progression of diabetic retinopathy.

##### ROS

Glucose oxidation makes the retina extremely susceptible to the generation of oxidized and NO_2_ species (ROS/RNS). Further, high tissue content of polyunsaturated fatty acids, oxygen uptake, and glucose oxidation make the retina extremely susceptible to the generation of oxidized and NO_2_ species (ROS/RNS) that result in modification of proteins, peroxidation of lipids, and DNA injury mainly from mitochondrial dysfunction in the involved neurovascular unit cells (50, 51). Furthermore, impaired antioxidant defense systems including reduced enzymes such as catalase and glutathione peroxidase and superoxide dismutase, also lead to generation of retinal ROS/RNS that are exacerbated by variations in tissue exposure to glucose (52, 53). Considering these conditions, it is not surprising that sleep apnea, with hypoxic episodes that result in retinal vessel dilation and hyperperfusion but are followed by reactive acute hypertensive reprise, are associated with severe exacerbation in the prevalence of diabetic retinopathy (54, 55).

##### Inflammatory cytokines and chemokines

Activation of Müller glial cells, a significant source of inflammatory modulators, may occur prior to clinical signs of diabetic retinopathy suggesting an early role in the onset of the inflammatory processes responsible for retinal damage noted at later stages of the disease (9) Inflammatory cytokines (e.g., TNF-α, IL-6, IL-8, IL1β, etc.) and chemokines (e.g., MCP-1, ICAMs) are upregulated in the serum as well as ocular samples of diabetes patients and are correlated with retinopathy severity (9, 56–59). Under hyperglycemic stress, microglia that are normally dormant macrophages located in the inner retina, become activated and with retraction of their dendrites become amoeboid and infiltrate deeper layers where they perform multiple inflammatory functions that can be either beneficial or harmful to the affected tissue but have been reported to contribute to neuronal damage by secreting neurotoxic factors with increased secretion of TNF-z, IL-6, and vascular endothelial growth factor (VEGF) (60). Later involvement of Müller cells and astrocytes, normally quiescent but supportive of the local environment for neuronal function in the healthy retina, results in the cells undergoing reactive gliosis discernible by upregulation of glial fibrillary acidic protein (GFAP) (3). Such reactive gliosis is associated with increased expression of VEGF and innate immune-related pathways that results in overexpression of proinflammatory cytokines producing an exacerbation of retinal microvascular leakage and eventually the proliferation of microvessels at the margin of ischemic areas that eventually stimulate their proliferation (3, 9, 18, 46).

##### Aberrant growth factor signaling

Multiple interconnected pathways (e.g., polyol, AGEs, protein kinase C, renin-angiotensin system, and hexosamine pathway) that are activated by diabetes increase the expression of inflammatory and angiogenic mediators thereby inducing aberrant growth factor signaling. This is directedly linked to the neurodegeneration of vascular dysfunction (9, 39).

Balance between upregulated and downregulated neuroprotective factors in the diabetic retina plays a key role in the health of the retinal neurons. In early stages of diabetic retinopathy, downregulation of key factors including PEDF, somatostatin, glucagon-like peptide 1 (GLP-1), and other neurotrophic factors is counterbalanced by an upregulation of VEGF (18). However, ultimately, the downregulation of neuroprotective factors may predominate, thus adding/contributing to retinal neurodegeneration (9, 18).

Hyperglycemia-induced oxidative stress plays major role in mediating both the expression and pathological effect of VEGF to aggravate the trapping of the leukocytes within the capillaries (53). In healthy patients, this process is normally inhibited by pigment epithelium-derived factor (PEDF), but in diabetic patients, there is a hyperglycemia-induced downregulation of a number of neurotrophic factors including PEDF (9). PEDF, a potent inhibitor of retinal vascular leakage and angiogenesis, is believed to protect the neurons from light damage and oxidative stress associated with abnormal autoregulation (9, 18, 61). In addition, activation of VEGF along with reduction in platelet-derived growth factor (PDGF) signaling is associated with loss of pericytes in the inner retina, as well as loss of synaptic activity and dendrite loss that result in apoptosis of neurons primarily in the ganglion-cell and inner nuclear layers (9, 24, 27, 28, 62). Furthermore, the oxidative milieu of the diabetic retina impairs processing of pro-NGF (believed to promote retinal neurodegeneration by activating pro-apoptotic pathways in retinal ganglion cells) to mature NGF (thought to prevent apoptosis of Muller glia and neurons) which contributes as well to ganglion cell death, increased vascular permeability, and inflammation (18, 63). Of note, neuronal apoptosis, which is now recognized to occur in the diabetic retina prior to documented vascular injury, shares many of the same mechanisms with the vascular damage (9, 18, 24).

##### Vascular dysfunction

Integrity of blood retinal barrier (BRB) is compromised by alterations in the neurovascular unit leading to changes in permeability of endothelial cells and surrounding pericytes of retinal capillaries that leads to increased secretion of cytokines and growth factors that result in low grade intra and extravascular inflammation with capillary basement membrane thickening, ICAM secretion and white blood cell obstruction and degradation (18). Indeed, the disruption of the blood retinal barrier is one of the most important events in the early stages of diabetic retinopathy (18, 46). The breakdown of the inner BRB results in the recognized microvascular leakage of the inner retinal vessels producing macular edema and lipid exudates, but there appears also to be an important role of the outer BRB as well (9). In both, a combination of capillary occlusion and increased capillary permeability result in the production of vascular leakage (9) due to the imbalance of the enzymes. The further generation of VEGF and downregulation of PEDF both contribute to the vascular dysfunction observed in severe diabetic retinopathy based on their ability to further promote vascular permeability and angiogenesis (9).

#### Genes, Environmental Factors, Epigenetic Changes

Hyperglycemia- and glucolipotoxicity-induced oxidative stress drives changes in chromatin structure that mediate gene expression changes resulting in upregulation of proinflammatory and profibrotic mediators (6, 64). These epigenetic changes are recognized as a key factor in the development and progression of vascular diabetic complications (64). Epigenetic modifications are generally produced by external changes (toxins, nutrients, etc.) that transduce inside cells to cause DNA methylation and histone modification leading to altered gene expression (6, 65). Non-coding RNAs (microRNAs) and long non-coding RNAs also regulate post-transcriptional gene expression (6, 65, 66). Such altered gene expression caused by epigenetic influences can occur as early as in the fetus within a pregnant diabetic but may accumulate throughout life with recurrent stress-induced influences (66, 67). Altered gene expression as a result of epigenetic changes leads to cell hypertrophy, proliferation, remodeling, and apoptotic signaling and are key factors for the development and progression of diabetic complications (6, 64, 65). Indeed, the expression of many genes implicated in metabolic pathways associated with the development of diabetic retinopathy have been found to be up or downregulated and changes to microRNAs expression playing key roles in diabetic retinopathy, including blood retinal breakdown and neovascularization expression, have also been observed (65).

Other environmental influences that may have a role in diabetes complications include the gut biome. Western diet, antibiotic use, and microbial exposure all have been reported to play a role in gut dysbiosis (68). It is hypothesized that this altered microbiome leads to reduction of GLP-1 secretion, short-chain fatty acid production, and low-grade inflammation and may also impact insulin resistance—all factors contributing to diabetes and its complications (6, 68). Indeed, studies have linked the microbiota to diabetic retinopathy (69). For example, restructuring of the gut microbiome by intermittent fasting was observed to prevent retinopathy in a diabetic mouse model (70). The authors hypothesized that the change in bile acid metabolism due to the restructured gut microbiome favored increased endogenous generation of TUDCA, a neuroprotective bile acid (with receptors demonstrated in retinal ganglion cells) offering a protective effect against injury (70). Such reactions appear to be related, not just to the overall average serum and tissue glucose, but also to variability in glucose concentration (71). However, additional research is warranted to further test if the microbiota is a risk factor for diabetic retinopathy.

#### Insulin Resistance, Abnormal Metabolic Environment, and Fuel Excess

Insulin resistance is associated with increased glucose production in the liver, decreased peripheral glucose uptake in the muscle, and increased lipolysis in the adipose tissue, all of which lead to β-cell dysfunction and the associated downstream effects of that dysfunction (section Diabetes and Its Complications Arise from Common Pathophysiologies) (6). The abnormal metabolic environment and fuel excess (gluco-lipotoxicity) within cells and tissues is further exacerbated by insulin resistance, inflammation, environmental factors, and genes/epigenetics through alteration of redox regulators and epigenetic modifications that then lead to cell dysfunction (6, 9, 32, 39, 41, 49).

In the case of diabetic retinopathy, metabolic dysfunction in the neuroglial unit leads to glial and endothelial cell damage in the retina (9, 18, 46, 49). This dysfunction leads to increased inflammation and ROSs and aberrant growth factor signaling resulting in neuro-glial degeneration and vascular dysfunction. This, in turn, is associated with capillary occlusion and microvascular leakage, alteration of the blood-retinal barrier, hypoxia, vascular permeability resulting in edema, and angiogenesis leading to the development/progression of diabetic retinopathy (9, 18, 72).

The impact of insulin resistance on risk for diabetic retinopathy is illustrated by the following examples. In an observational matched cohort study of Type 2 diabetes patients, insulin resistance was an independent and specific risk factor for proliferative retinopathy (73). In Type 1 diabetes patients, estimated insulin sensitivity was associated with lower odds of developing diabetic retinopathy and proliferative diabetic retinopathy in a prospective, longitudinal, observational study (74).

In addition, dyslipidemia and hypertension also influence DR with recent data strongly supporting the fact that dyslipidemia plays an important role in the development as well as progression of diabetic retinopathy (5, 75, 76). Furthermore, insulin resistance, dyslipidemia, and hyperglycemia collectively (metabolic syndrome) are thought to drive diabetic retinopathy in patients with metabolic syndrome (a syndrome consisting of three or more of the following traits: large waist, high triglyceride level, reduced HDL cholesterol, increased blood pressure, and elevated fasting blood glucose), with and without a history of established diabetes suggesting inflammation, genes, and environment may potentially have important roles causing retinopathy often before hyperglycemia is detected in an individual (77).

## Implications for Diabetic Retinopathy Diagnostic Screening and Treatment

Meaningful treatment of diabetic retinopathy has been limited by failure to recognize the neurovascular condition which precedes and then continues in parallel with the progressive microangiopathy (18–23). For example, although anti-VEFG therapies are initiated for treating all manner of retinopathy with observed retinal central thickening, they only result in modest, and very often transient, improvement of visual acuity (10–12).

With this in mind, approaches to vision screening should be modified. For example, in contrast to visual examination using a high-contrast chart (black letters against a white background), central vision loss can be detected under conditions that simulate mesopic, low illumination, or glare environments prior to observable or clinical retinopathy (29, 30). Thus, the screening and entry criteria for the consideration of treatment, previously initiated by physician examination of the retina and measurement of vision with the high contrast letter chart, should cease (13, 29). In addition, inner retinal ganglion cell changes with reductions in nerve fiber layer thickness can be observed by spectral domain optical coherence tomography (SDOCT) with progression of the thinning equal in many cases to that observed in glaucomatous eyes (18, 78–84). Prospective studies using new technologies are appearing (e.g., low illumination and low contrast resolution perimetry, OCTa-based oximetry with digital microvascular integrity analysis along with scanning laser ophthalmoscopic imaging of neuronal apoptosis fluorescent tags). Such techniques should provide improved evaluation of retinal injury to both the neuronal unit as well as the microvasculature and allow for improved, earlier treatments, both local as well as systemic. Currently there are no systemic factors established for retinopathy risk screening outside of hyperglycemia (HgbA1c), glycemic variability, hypertension, and obstructive sleep apnea, although early reviews suggest some serum autoimmune inflammatory constituents (85) and circulating miRNA markers are associated with early stages of diabetic retinopathy (86), and certainly need to be further investigated for screening and to define chronologic progression.

Approaches to treatment and prevention should recognize that significant alterations and inflammatory injury occur to both the neuronal unit as well as to the microvasculature earlier than once thought and that better methods for both early detection and treatment are warranted.

### Current Treatments and Approaches

Current treatments for diabetic retinopathy include laser treatments, intravitreous injections of anti-VEGF and steroid agents, vitreoretinal surgery, and glucose control (Table 1). However, these approaches are often not begun until the patient presents to the eye doctor with vision problems that impair daily living. As explained above, contemporary screening methodologies and diagnostic criteria used to initiate treatment are severely limited such that treatment is initiated far too late, after the neurodegeneration has irremediably progressed. Mild retinopathy without edema is very often not treated at all when associated with moderately good chart visual acuity.

**Table 1:** Current treatment and approaches to diabetic retinopathy.

**Target**	**Therapy**	**Key Considerations/Information**
**Local treatments**		
Peripheral ischemia associated with neovascular proliferation	Laser treatments Panretinal laser photocoagulation therapy Focal/grid laser	Laser photocoagulation has been applied in a diffuse, scatter mode to treat peripheral ischemia associated with neovascular proliferation with improvement in the adverse events of vitreous hemorrhage and traction detachment Reduce risk of severe visual loss in proliferative diabetic retinopathy, inhibit progression of diabetes retinopathy Reduce risk of moderate vision loss, increases changes of visual improvement, decrease frequency of persistent macular edema Risk for visual acuity loss, visual field loss, and constriction of visual field has lead to increased use of subthreshold, micropulsed laser application, or intravitreal anti-VEGF injections
Vitreous hemorrhage Retinal detachment	Vitrectomy Cryotherapy	Used in cases of proliferative diabetic retinopathy with long-standing vitreous hemorrhage, tractional retinal detachment, or combined tractional and rhegmatogenous retinal detachment Cryotherapy is used when laser photcoagulation is unacceptable due to an opaque media (e.g., cataracts or vitreous hemorrhage)
Inflammation	Intravitreal steroids DEX implant (Ozurdex) FA insert (Retiser, Iluvien) Triamcinolone—off label Anti-TNF-α (infliximab, adalimumab)—off label	Used for elevation in intraocular pressure, vitreous hemorrhage, glaucoma, cataract surgery Used increasingly in the treatment of diabetic macular edema refractory to anti-VEGF therapy alone FDA approved indications in gastroenterology (Crohn's disease, ulcerative colitis) rheumatology (rheumatoid arthritis, psoriatic arthritis, ankylosing spondylitis), and dermatology (plaque psoriasis)
VEGF	Intravitreal injections of anti-VEGF antibodies Aflibercept (Eylea) Ranibizumab (Lucentis) Pegaptanib (Macugen) Bevacizumab (Avastin)—off label	Reduce diabetic macular edema and neovascularization of disc or retina (used in advanced diabetes retinopathy stages) Associated with a modest, visual acuity improvement with a high proportion of non-responders and with a substantial number of others developing resistance over time, with retinal atrophy, and suboptimal visual acuity after 2–5 years of anti-VEGF treatment Of note, anti-VEGF treatments may impact inflammation as well as angiogenesis as VEGF overexpression is associated with overexpression of proinflammatory cytokines which leads to exacerbation of retinal microvascular leakage and proliferation of microvessels
**Systemic treatments**		
Hyperglycemia	Traditional approaches to treatment of hyperglycemia	Intensive glucose control in patients with Type 1 diabetes decreased incidence and progression of diabetic retinopathy. The same principals are thought to apply to other DM types However, treatment with insulin, and resultant hyperinsulemia, is likely associated with adverse outcomes (e.g., CV risk, weight gain, obesity, endothelial dysfunction, atherosclerosis, hypertension, chronic inflammation, and cancer)

*Bhavsar (87), Bressler et al. (88), Brown et al. (10), Duh et al. (5), Reddy et al. (12), Rübsam et al. (9), Schwartz et al. (89), Schwartz et al. (6), Simo et al. (22), Simo et al. (18), and Wang and Lo (90)*.

### Expanding the Armamentarium—Broadening Our Approach to Treatment of Diabetic Retinopathy

Systemic as well as localized retinal directed treatments must be pursued with adoption of an attitude of “predict and prevent” rather than waiting for and then attempting to treat only after severe neuronal and vascular injury has occurred. Furthermore, there is the need for an expansion of our current thinking to evolve our approaches to treatment to be aligned to what is now known regarding diabetes and its complications as influenced by the multiple interrelated pathophysiologic mechanisms that impact cell and tissue damage within the retinal neurovascular unit leading to glial, neural, and microvascular dysfunction. To wit, an adverse metabolic environment engenders increased pathways described by Brownlee which lead to generation of ROS which in turn leads to increased inflammation and epigenetic changes (39). Therefore, by targeting the fuel excess and inflammation more broadly, one is able to impact diabetic retinopathy with a multifaceted approach.

This method of thinking allows for the potential of preventing, treating, or delaying diabetic retinopathy progression with agents used for glycemic control that have additional impact on mechanisms contributing to complications as well as agents aimed specifically at pathophysiologic mechanisms driving diabetes complications (e.g., inflammation, insulin resistance, etc.) (6, 20, 44, 45). One example of the potential to use an agent indicated to reduce hyperglycemia in patients with diabetes, but also has other beneficial impact on complications is glucagon-like peptide-1 (GLP-1) receptor agonists. GLP-1 agonists are indicated for improving glycemic control and reducing risk of major cardiovascular events in diabetes patients with established CV disease by impacting incretin regulation and insulin resistance (44, 91). In a diabetic retinopathy rat model, topical administration of GLP-1 reverted the impairment of the neurovascular unit by exerting anti-inflammatory action, decreasing VEGF expression, promoting cell survival, and inducing neurogenesis (92). This intriguing data along with our new understanding of the multiple pathophysiologic mechanisms contributing to diabetes and its complications suggests that GLP-1 agonists could be beneficial in slowing and or preventing diabetic retinopathy in patients. Other promising “diabetes” agents that impact hyperglycemia through multiple routes and which may be beneficial in the treatment of diabetic retinopathy include metformin, DPP-4, and SGLT-2 inhibitors to name a few but are still unproven (44, 45).

Furthermore, approaches for prevention or treatment of diabetic retinopathy expand even beyond the use of agents used for glycemic control with additional impact on other comorbidities and mechanisms of β-cell damage. Some of these potential approaches are discussed below. It is important to note, that further research is warranted to provide evidence to support their use specifically for diabetic retinopathy.

#### Addressing Oxidative Stress and Epigenetic Changes

As discussed earlier (sections Diabetes and Its Complications Arise from Common Pathophysiologies, Retinopathy Evidence Organized by Pathophysiologic Mechanism), oxidative stress and ROS lead to inflammation, and the induction of transcription factors that result in altered gene expression and epigenetic changes. This in turn leads to cell dysfunction, hypertrophy, proliferation, remodeling, and apoptosis (6, 39, 40, 44).

Thiamine (vitamin B1) is an essential cofactor required at several stages of energy metabolism including intracellular glucose metabolism (93, 94). Benfotiamine, a synthetic S-acyl thiamine precursor with higher bioavailability than thiamine, reduces flux through four major pathways of hyperglycemia damage (AGE, hexosamine, protein kinase C, and polyol pathways) and has been shown to prevent diabetic retinopathy and nephropathy in experimental *in vitro* and *in vivo* models (93–95). In clinical studies, benfotiamine has been shown to prevent the development of diabetic neuropathy (93). However, excess superoxide/ROS can damage cells and tissues independently of these pathways. Therefore, Du et al. have proposed adding α-lipoic acid, an antioxidant, to benfotiamine, to address the four major pathways of damage as well as independent additional damage from ROS (95). In a pilot study of this combination treatment in Type 1 diabetic patients, normalization of several complication-causing pathways was observed including reducing AGE formation, reducing hexosamine-modified proteins, and normalizing oxidative inactivation (95). Further trials appear warranted to evaluate the potential of this combined approach to prevent and/or treat diabetic retinopathy.

#### Treatment of Inflammation

The inflammatory process, as discussed above, plays an important role in the pathogenesis of diabetic retinopathy including increased retinal vascular permeability, occlusion, neovascularization, and retinal neurodegeneration (9, 45, 47, 59). Thus, consideration of treatments directed at inflammation should be added to physician's tool kit to treat, manage, or prevent diabetic retinopathy.

There is accumulating evidence that targeting pro-inflammatory cytokines and chemokines (beyond VEGF) may have benefits for the management of diabetic retinopathy (96). Indeed, TNF-α inhibitors are used off label to treat diabetic retinopathy. However, despite evidence linking TNF-α to inflammation and increased permeability in diabetic retinopathy, results of studies of treatment with systemic TNF-α inhibitors have been mixed (96, 97). For example, Wu et al., have conducted an open-label uncontrolled multicenter retrospective study of 39 eyes with refractory diabetic macular edema in Type 2 diabetics treated with intravitreal infliximab or adalimumab (98). In the infliximab group, the logMAR chart acuity did not improve at 3 months or worsened with minimal change in the central retinal thickness. With adalimumab treatment, the logMAR chart acuity improved more substantially (nearly 0.33 logMar) but with virtually no change in the central retinal thickness. In review, it appears that these mixed results with the systemic TNF-α inhibitors were perhaps likely due to the fact that the systemic drugs only weakly crossed the blood-retinal barrier (96, 97).

More recent approaches have examined other targets, mainly IL-1 and IL-6, with preliminary encouraging results reported with anakinra, canakinumab (selective IL-1b antibody), and tocilizumab (9, 99). For example, in a pilot study, canakinumab showed stabilization of retinal neovascularization and macular edema reduction in patients with proliferative diabetic retinopathy (9). Furthermore, early studies of an inhibitor of atypical protein kinase C appear also to prevent TNF-α as well as VEGF-induced permeability, providing a broad target for potential control of edema (100, 101).

In recent studies of diabetic animal models, peptide derivatives of PEDF, hypothesized to have a multifunctional role preventing retinal inflammation, vascular hyperpermeability, and neuronal dysfunction through reduction of oxidative stress and glutamate excitoxicity, delivered in the form of eye drops, were observed to reduce neurodegeneration, vascular permeability, and inflammation in mouse retinas (61, 102–104). Clinical trials have begun (61). In addition a recent trial of diabetes patients with no, mild or moderative non-proliferative diabetic retinopathy demonstrated preserved visual function compared with placebo after a 6-month course of anti-oxidants (96).

NSAIDs, in addition, are being investigated for the potential to mitigate the progression of early diabetic retinopathy (96). For example, ketorolac, coxib, nepafenac, and dicofeac have been investigated as possible treatments for diabetic macular edema. In retinal capillaries, endothelial ICAM expression, and nuclear factor κB activation, which produce leukocyte adhesion and capillary obstruction, were normalized by treatment with NSAIDs (cyclooxygenase-2 inhibitor, meloxicam, or a soluble TNF-α receptor blocker—etanercept) in early diabetic retinopathy animal models (105, 106). In addition, pentoxifylline, improves both ICAM development and leukocyte deformability, and appear to improve blood flow in the human diabetic retina but it is unclear if this leads to a significant impact on diabetic retinopathy (107, 108). These findings suggest further studies are warranted to evaluate their overall effect on the vascular component of the retinopathy. It is thought that NSAIDs may represent a promising therapy for reducing inflammatory mediators without the risk of cataracts and elevated intraocular pressure associated with corticosteroids (96).

Angiopoetins, inflammatory growth factors, bind to the receptor tyrosine kinase Tie2 and are considered important regulators of blood-retinal barrier (9). Accordingly, a number of Tie2 activator drugs injected intraocularly are under investigation. Subcutaneous injections of a Tie-2 activator, AKB-9778 in combination with anti-VEGF therapy in phase 2 clinical trials for DR demonstrated significantly reduced diabetic macular edema and improved visual acuity compared to anti-VEGF alone (9, 109).

In review, it appears that treatment for retinopathy and other systemic outcomes requires a multifactorial approach to address the more intimate mechanisms responsible for the large as well as small vessel disease and inflammatory destruction of the tissue. Perhaps by combining various anti-inflammatory drugs, broader and more efficacious therapeutic strategies may be developed. For instance, some anti- inflammatory treatments (such as anti-IL-1β agents) seem to be more effective at improving insulin secretion, whereas others (i.e., the anti-TNF agents and salsalate) may primarily affect the target, insulin-sensitive tissues (99). To examine this hypothesis, the planned INFLACOMB trial has the ambitious goal to compare various anti-inflammatory strategies in multiple combinations (99).

#### Treatment/Prevention of Neurodegeneration

In addition to the recognition and treatment of the inflammatory process that injures the neurons along with the microvasculature, progress should entail the consideration of treatment that prevents neurodegeneration since substantial reversal, once the degeneration has progressed, is limited. Certainly, with recognition of such neuropathic injury occurring in the retina, retinal treatments can and must be pursued earlier, but with consideration of those which can be easily applied/used with minimal adverse effects.

Neurotrophins trigger neuroprotective signaling cascades and one such factor, brain-derived neurotrophic factor, has been shown to protect ganglion, glial, and amacrine cells from death in diabetic rats (110, 111). Activation of the molecular chaperone, sigma 1 receptor, in the retina is another promising therapeutic target for retinal degenerative diseases. Sigma 1 receptor activation in a mouse model of retinal degeneration has been shown to protect against ganglion cell loss and preserve cone photoreceptor function (112). Furthermore, imbalance in levels of native neuroprotective factors in the retina such as somatostatin, insulin, PEDF, and ciliary neurotrophic factor (CNTF) can also be corrected by supplementation (61, 113–115). Moreover, topical administration of the neuroprotective drugs brimonidine and somatostatin appears to be useful in mitigating the worsening of preexisting retinal neurodysfunction in patients with diabetes (116).

Topical administration of GLP-1 and DPP-4 inhibitors prevented retinal neurodegeneration and vascular leakage in rodent models (92, 114, 117) The effect of DPP-4 inhibitor was thought to be due to prevention of GLP-1 degradation. Of note, GLP-1 retinal levels are significantly lower in patients with diabetes compared with controls (114).

Endothelin-1 (ET-1), upregulated in the retina of patients with diabetes, is also an alternative target for prevention of neurodegeneration in diabetic retinopathy (118). ET-1 has dual deleterious actions on microvessels and neurons caused by its capacity to bind to endothelin receptors A (ETA) which mainly mediate vasoconstriction and vascular degeneration and B (ETB), involved in retinal neurodegeneration (119). Therefore, the blockade of such ET-1 derivatives may offer a means to prevent both diabetes induced microvascular disease and neurodegeneration, but is yet to be investigated.

Animal studies have demonstrated the presence of erythropoietin receptors on the endothelium of multiple organs that produce an independent vascular tissue protective effect of systemic recombinant erythropoietin treatment within the brain, spinal cord, peripheral nerves, retina, heart, kidney, and intestine (120). Within these tissues, hypoxia appears the primary regulator of erythropoietin and erythropoietin receptor transcription although other stimulating factors/conditions (activation of IL-6 downstream signaling pathway) are also involved in the upregulation of erythropoietin and receptor production within the diabetic retina (121–123). Neuroprotective effects of intravitreal erythropoietin injections have been seen through anti-apoptotic, antioxidant, and anti-inflammatory actions in animal models (122, 124–126). Systemic use of erythropoietin has been associated with side effects including hypertension and increased risk of thrombosis, thus early, small clinical trials have investigated intravitreal delivery of recombinant EPO (118). In a small FDA phase 1a safety and tolerability trial, intravitreal erythropoietin (Procrit) in five eyes of five patients that were unresponsive to prior surgery, laser, steroid and anti-VEGF demonstrated a logMAR 0.3 or more significant improvement in chart acuity and central field acuity perimetry, with clearing of exudates, but little change in leakage defined by fluorescein angiography at 16 weeks ongoing injection and follow-up (127). Ongoing clinical trials are in progress and appear warranted (128).

With regard to other local, ocular treatments that exhibit potential effects toward treatment or prevention of retinopathy, more recently the application of a new mode of non-thermal, high-density micropulsed retinal laser treatment has been introduced that has demonstrated in animal studies to produce anti-inflammatory and neuro-reparative cytokines that improve retinal pigment epithelium function, as well as retinal vascular autoregulation, while reducing the markers of chronic inflammation, and neurodegeneration (129, 130). The generated reparative enzymes, formerly termed “heat shock proteins” that resulted from retinal pigment epithelium non-lethal laser heating that have thus far been measured include reductions in thiobarbituric acid reactive substances (TBARS), increased glutathione (GSH) and superoxide dismutase 1 (SOD1) as well as a reduction in cytochrome c, caspase 3 expression and activity along with cleaved caspase 9, and increased Beclin 1, p62, and LC3b (131). Reductions in markers of inflammation have also been documented in human diabetic retinopathy (132, 133), and micropulsed laser treatment has been demonstrated to improved early forms of macular edema with significant improvement in vision and without evidence of laser injury (133–135). When the macular edema is more severe, however, similar to the studies of intravitreal anti-VEGF injections, the studies of micropulsed laser demonstrate only minimal improvement in the edema and in vision (presumably because of the already established, irreversible neurodegeneration) (11, 133). Studies are underway to investigate the ability of this non-invasive treatment to retard the progressive neuronal apoptosis and vision loss and progression of the microangiopathy when treatment is applied early and repeatedly.

#### Lipid Metabolism Regulation

In older type 2 diabetics, hyperlipidemia, occurring with hypertension, has been demonstrated associated with worse stages of retinopathy and with worse accumulation of intra-retinal lipid exudates (136). Due to the critical role of dyslipidemia in the progression of diabetic retinopathy, multiple potential therapeutic targets are under investigation (75).

While statin use is associated with improvement in such retinal exudates, systemically it is also associated with a lower degree of myocardial microangiopathy and systolic dysfunction in patients with dilated cardiomyopathy (137). Use of the PPAR-antagonist, fenofibrate, has been demonstrated to reduce the risk of progression of non-proliferative diabetic retinopathy by up to 40%, but whether this is related to the lipid lowering or other effects is unclear (138, 139). In the retina, however, essential omega-3 long-chain polyunsaturated fatty acids (PUFA's) cannot be synthesized in sufficient amounts by humans and must therefore be obtained from diet. The retina has the highest omega-3-PUFA concentration of all tissues and in normal retinal architecture these exhibit neuroprotective actions (140). Docosahexaenoic acid (DHA), a major dietary omega-3-PUFA and a major structural lipid of retinal photoreceptor outer segment membranes, is the precursor of NPD-1, a docosatriene that is required for the functional integrity of the retinal pigment epithelial protecting these cells from oxidative stress, has an antiapoptotic effect. These fatty acids have the potential to treat diabetic retinopathy through a wide range of properties including anti-inflammatory, anti-oxidant, and anti-angiogenic effects (141). However, a specific clinical trial designed to address this hypothesis is still in need (141).

## Conclusion

Diabetic retinopathy is now recognized to be an inflammatory neuro-vascular complication of the systemic disease with neuronal injury/dysfunction preceding the current clinical microvascular recognized damage and furthermore, is indicative of the inflammatory tissue injury concurrent in other organs.

The current diagnosis and treatment for diabetic retinopathy are insufficient. Contemporary screening methodologies and diagnostic criteria used to initiate treatment are severely limited such that treatment is initiated far too late, after the neurodegeneration has irremediably progressed. These screening techniques consisting of physician examination, white light photography, and high contrast chart acuity must cease for the screening as well as for evaluation of treatment in favor of diagnostic modalities that assess the structural and functional vision analysis of neuronal destruction that will indicate injury earlier in the course, predict the progression, and monitor the impact of both ocular as well as systemic treatments.

Currently, there are limited treatment options for diabetic retinopathy. Viewing diabetic retinopathy within the context whereby diabetes and all its complications arise from common pathophysiologic factors allows for the consideration of a wider array of potential ocular as well as systemic treatments, for this common and devastating diabetes associated complication. However, it will be important to further study these potential approaches and such that evidenced-based treatment decisions can be made for patients with diabetic retinopathy.

Diabetic retinopathy is an inflammatory neuro-vascular complication with neuronal injury/dysfunction that preceds clinical microvascular damage. This cellular and tissue damage in the neovascular retinal unit is due to similar pathophysiologic factors implicated in the damage to pancreas β-cells and other organ injury. Agents used for the treatment of diabetes must address these recognized pathophysiologic inflammatory mechanisms (inflammation, epigenetic changes, insulin resistance, fuel excess, and abnormal metabolic environment) that have been identified in other organ injury that parallels the retina. The retina, because of the ease of functional and structural investigation, offers the means to assess the inflammatory destructive disease process in the individual as well as to prove the efficacy to prevent and mitigate damage, not only to retinal tissue and vision but the parallel processes in the other organs as well. This approach adds to the physician's armamentarium and increases the opportunity for prevention and early treatment of diabetic retinopathy and other complications as well.

## Author Contributions

SHS and SSS contributed to the conception of the work, drafting the work as well as critically revising it for important intellectual content, read, and approved the submitted version of the manuscript.

### Conflict of Interest

In the commercial interests both SSS and SHS state that they are employed and receive financial compensation only from their clinical practices, SHS's being Sinclair Retina Associates, SSS's being Stanley Schwartz, MD, LLC [affiliated with Main Line Health (with no monetary connection)] with no compensation related to the manuscript. Through SinclairTechnologies, SHS owns the rights to the Omnifield and Central Vision Analyzer that are mentioned in the paper along with a patent on the intraocular use of erythropoietin, but these have not manifested in financial gain. SSS is on advisory boards for Salix Pharmaceuticals and Arkay Therapeutics and on the Speaker's Bureau for Salix Pharmaceuticals, Janssen Pharmaceuticals, Boehringer Ingelheim, Eli Lily, and Merck.
